# Insect-flower interaction networks vary among endemic pollinator taxa over an elevation gradient

**DOI:** 10.1371/journal.pone.0207453

**Published:** 2018-11-29

**Authors:** Opeyemi A. Adedoja, Temitope Kehinde, Michael J. Samways

**Affiliations:** 1 Department of Conservation Ecology and Entomology, Stellenbosch University, Stellenbosch, South Africa; 2 Department of Zoology, Obafemi Awolowo University, Ile-Ife, Nigeria; RMIT University, AUSTRALIA

## Abstract

Interaction networks are sensitive to elevation gradients through changes in local distribution of interacting partners. Here, we use plant-pollinator interaction network metrics to assess the effect of elevation on flowers and flower-visiting insect assemblages on a sentinel mountain used for monitoring climate change in the flower- and insect-rich Cape Floristic Region. We also use these interaction metrics to explain the effect of environmental factors on the interaction networks. We did this over four vegetation zones <1640m asl, as determined by former botanical studies. Overall, bees were the dominant flower visitors, followed by monkey beetles, and far behind were wasps and flies. The middle elevation zone (650–744 m a.s.l), which is also an ecotone between two distinct botanical zones, had the highest species richness and abundance of interacting plants and insects. Interaction frequency and size of network were also greatest in the middle zone, as were network diversity, generality, and linkage density, while lowest in the peak zone. In sum, there was distinct elevation zoning of flower-visiting insects. The greatest zonal change was between species at the middle compared with peak zone. Large-sized monkey beetles, bees and flies characterized the unique assemblage in the peak zone (1576–1640 m a.s.l.). The insect zonation tracked that of plant assemblages, with air temperature (lapse rate) being the primary driver of bee distribution, with lowest levels in the peak zone. In contrast, beetle distribution was driven mostly by flower assemblages as well as air temperature. In turn, wasp and fly interaction networks were not affected by any of the measured environmental variables. We conclude that increased elevation stress from reduced temperatures, changing abiotic weather conditions (e.g. strong winds at high elevations),and decline in flowering plant composition causes breakdown of interaction networks involving bees and beetles but not that of flies and wasps.

## Introduction

Plant-pollinator interaction networks are valuable for assessing biodiversity change and landscape quality in response to stressors [1]. Changes in these networks also lead to changes in the interaction metrics, most of which are defined by interaction frequency. However, careful analysis and interpretation of these metrics are important for identifying particular stressors on communities [2]. As there are several network metrics used to interpret stressors, it is necessary to identify the ones that best explain specific patterns of change in interaction networks.

Natural ecosystems change across environmental gradients, as well as from turnover of mutualistic relationships among species [3, 4]. Changes in mutualistic interactions, such as plant-pollinator interactions across latitudinal gradients, leads to interactions in the tropics being more specialised through high species diversity compared to that in temperate regions [5]. Community composition and mutualistic interactions also respond to changes across elevation gradients, which illustrate how environmental stress influences biotic communities [6, 7]. Responses of bee-plant interactions across elevation gradients have been explored [8, 9]. However, little information is available on bees compare to other pollinator taxonomic groups such as beetles, wasps and flies as regards their response to elevation gradients, especially in Africa.

Elevation gradients are an important component of many natural landscapes, and can greatly affect environmental variables, even over short range [10]. These gradients provide opportunities for studying biotic responses to changes in air temperature (the lapse rate), precipitation, solar radiation, soil properties, reduced land area, and other abiotic features of montane ecosystems [11, 12,13]. With every 100 m increase in elevation, there is a drop of 1^°^C in air temperature [14], resulting in delayed growth and flowering of plants as well as their reproductive success. This in turn, influences their insect visitors [15, 16]. Reduced productivity often affects the flower-visiting insects more than the plants [17], which has a cost implication in the context of mutualisms such as pollination, and also partner specialization within interaction networks[18, 19, 20, 21].

Plant-pollinator interactions are sensitive to abiotic conditions that affect the interacting partners. Distribution of plant and insect pollinators across an elevation gradient determines pollination success, especially as there is often an increase in frequency and intensity of adverse weather with increasing elevation. With the exception of adaptive species which show higher flower longevity in harsh conditions at peak elevations [22], the warmer conditions at low elevations allow longer flowering times, as well as affecting the local distribution of various insect species [23, 24]. This low-elevation effect positively affects mass flowering of some plant species [25], with experimental warming in the arctic increasing reproductive success of flowering plants through an increase in number of flowers [26, 27].

There are few studies on how insect pollinator taxa are differentially affected across elevation gradients with their differing environmental conditions. However, there is some information on the differential effects of weather on various insect groups. For example, cold and wet weather positively influences the distribution of flies, while bees respond better to warm and dry conditions [28, 29, 30, 31]. The response of different groups also depends on life history traits [32, 6, 33], including sociality, nesting behaviour, body size, reproduction pattern, diet requirements etc. Higher insect sociality is usually an attribute of warmer low elevations [33], with voltinism depending on length of season and time of appearance of flowers, both of which decline or change with decreasing temperatures associated with increasing elevation [34].

Availability of interacting partners at various elevations is an important determinant of the types of mutualistic interactions present. The local presence or absence of interacting partners is determined by temperature across the elevation gradient, as well as the respective, innate ability of the flowers to produce floral resources and the insects to pollinate at the various temperatures, as well as under particular weather conditions. Increase in temperature beyond the thermal tolerance level of a biotic community results in an upward shift along the elevation gradient to a cooler region [35, 36]. Declines in abundance of ants and plants at high elevations results in fewer interacting partners high up, due to reduced richness and abundance of plant and ant species. At high elevations, there are fewer interactions, encouraging more connected networks, where they are less specialized [37].

The bee fauna of the Cape Floristic Region (CFR) is among the most vulnerable to increased global warming due to the high level of endemism of most species, as well as the small size of this region in the southwestern tip of Africa [38]. Bees, like most other pollinator taxa, are dependent on the environmental temperature for their activities. This may be a critical factor associated with foraging activities, body size at maturity, and the insect’s life span [24]. Large-bodied bees, such as xylocopids, megachilids and apids, are capable of generating internal heat to optimize foraging activities, even when the environmental temperature is low [39]. However, smaller bees such as *Lassiglosum* spp. have to hibernate to avoid colder habitats during a temperature drop below thermal tolerance [40]. In Wyoming, USA, large bumblebees have high tolerance for low temperatures of about 1^°^C at a high elevation of 3290 m asl. Conversely, bumblebee species at lower elevations are smaller, and have reduced tolerance to extreme temperatures [39].

Elevation has been used to assess the effect of climate change on pollinators [41]. However, little is known about how elevation influences plant-pollinator interaction networks. Most studies have been in the northern hemisphere with its history of glaciation events, while there are no studies yet in southern Africa which has had no glaciation for >200 myr. This is an important knowledge gap for a biodiversity hotspot like the CFR, where it is predicted that there will be a change in plant communities through a rise of 1.8^°^C by 2050 [42].

We aim here to determine how different groups of insect pollinators and their interactions respond to changes in abiotic conditions with elevation. We hypothesize that:(i) species composition of flower-visiting insects will vary across the elevation gradient, (ii) change in flower-visiting insects species composition will track changes in flowering plant communities, (iii) flowering plant diversity and area of floral display will be the most important factor predicting changes in insect species composition, and (iv) network properties will change with elevation, with more nested networks at lower elevations.

## Sites and methods

The study was conducted on Jonaskop Mountain (33°58'10.67''S, 19°30'21.96''E), Western Cape Province, South Africa, in the Cape Floristic Region biodiversity hotspot with a research permit from CapeNature. The bee diversity of the CFR is exceptionally high, coinciding with that of plants [43]. Prior to the commencement of the field work, we received a research permit from CapeNature. Jonaskop Mountain, our focal study area, is 1640 m high, and supports many localized sclerophyllous fynbos plant species. The mountain has distinct vegetation zones, and is a sentinel mountain for recording climate change [44].

Our study sites with increasing elevation on the mountain were based on previous vegetation profiling [45]. Lower elevations (< 550 m asl, 33°55'03.8''S, 19°30'46.1''E, ‘Base zone’) are characterized by succulent karoo, with 80% of the plant species at these elevations being endemic to the mountain. Elevations 650–744 m asl (33°55'28.2''S, 19°30'59.4''E, ‘Middle zone’) are an ecotone between the lower elevations, and the third zone (33°57'06.5''S, 19°31'02.0''E‘High zone’), characterized by Mid-elevation Sandstone Fynbos at 953–1303 m asl. The peak elevation (>1576 m asl, 33°58'09.0''S, 19°29'45.3''E, ‘Peak zone’) is classified as High-elevation Sandstone Dwarf Fynbos [46].

Plant-pollinator interactions were recorded at 18 sites within each of the four zones of the mountain August-October 2017, the peak flowering season. Each site was a 50 m^2^ plot, and plots within any one zone were 100–500 m apart. Groups of these sites, representing the four zones, were 0.8–2 km apart. Observations were done fortnightly at each zone, with plant-pollinator interactions conducted during five visits to each zone, except the peak, where the short flowering period permitted only three visits.

Timed observation of insect activity was standardized to 10 min/2 m^2^ plot to avoid over-emphasizing the specialization of flowering plants [47]. During this time, an interaction was noted when an insect visited the floral unit of a plant. Flower-visiting insects were identified in the field, or caught for later identification. Five replicates per 2 m^2^ sampling unit, made a total of 50 min total observation time per site for each visit.

Flower abundance of each plant species was estimated in each 2 m^2^ plot where insect activities were observed. A flower unit was defined here as the unit from which a honeybee-sized insect will fly to the next unit rather than walk [48]. Area of floral display was determined for each open flowering plant species by measuring the diameter of 1–10 flowers per plant species. Areas of flowers with circular outline was estimated using *πr*^2^ and L x B for rectangular surface outline flowers. A flower with visible depth, such as that of *Protea repens*, was estimated using 2 *πr*^2^*d* + *πr*^2^. The mean flower area for a plant species, together with the total abundance of flowers, was used to estimate the plant flower area per site [49].

Ambient air temperature was measured at each sampling period at the height of the flowers. Plant indices included flowering plant species richness, estimated by counting number of flowering plant species per site. The Shannon diversity index, which takes into account the flower abundance and richness, was used to estimate flowering plant diversity.

### Statistical analyses

Web structure for plant-pollinator interaction networks was computed for each site visit. Eighteen interaction web structures were computed using the plotweb command in the bipartite package in R [50]. Network level function was also used to compute the network metrics and species level analysis for species specialisation. Network qualitative properties, such as species richness and abundance of flower and insects, were also computed.

We compared number of interactions, network size, and flower-visiting insect species richness across elevation zones using generalized linear mixed effect models. We applied the “glmer” function and specified “poisson” family for our data. To account for overlap between sampling of each zone, we included the sites as a random variable in our model. We computed the square-root for flowering plant species richness and we included this in a general linear model (glm) to compute differences in plant richness across zones.

Network metrics, such as connectance, network nestedness, linkage density, network specialization (H_2_’), network generality, and network interaction strength asymmetry were computed using the network level command in the bipartite package.

Definitions used here are as follows: 1) Connectance: the proportion of realized interactions out of all possible interactions in a network [51], 2) Generality: explains the number of flower resources of a plant species available for an insect species in the interaction network [52], 3) Nestedness: describes the ability of specialist species in the network to interact with the species that also receive interactions from most generalised species in the network [53], and ranges from 1 to 100, and usually confers stability to interaction networks where the higher the nestedness value, the more stable and resilient the network is to disruption [54], 4) Network specialization (H_2_’):estimates the selection and constancy of interaction between partners in a network by calculating the deviation of observed interaction from the expected null frequencies of interactions [55], and ranges from 0 (generalized network) to 1 (perfectly specialized network), 5) Species level of specialization (d): describes the deviation of observed visits to expected visits based on interaction of a focal species of insect in a network, and is determined by the availability of floral resources [56], 6) Linkage density: describes the degree of distribution of interacting partners in a network, and takes into account species richness and evenness of the distribution. Linkage density may be a better descriptor of network stability compared to nestedness of a network, but this is only the case for large networks [57], and 7) Interaction strength asymmetry (ISA): the strength and degree of interaction between partners is not usually the same in a network, which means that the effect of an interaction between an insect and a flowering plant is not the same as the effect of interaction that the plant has with the insect. This metric helps to understand the mismatch in the effect that a species has on interacting partner, and is reciprocal in an interaction network [58].

Network indices were log transformed to fit into normal distribution, and data compared among zones using simple Analysis of Variance (ANOVA). The relationship between number of interactions of each of the taxonomic groups with flower diversity and area of floral display was computed using Spearman rank correlation. Difference in composition of flower-visiting insects and flowering plant species among zones was estimated using Bray Curtis distance between zones in Primer v6. Analysis of Similarity (ANOSIM) was then computed to determine the degree of separation or similarity of interacting species between zones of elevation. Principal Component Ordination (PCO) was also computed to visualize the separation of insect activities across elevations.

The effect of area of floral display, flower richness, flower diversity, flower abundance, and temperature on frequency of interaction were computed using the Distance based linear model (Distlm) in Primer V6. Stepwise selection regression, together with Alkaike information criterion (AIC), were then used to assess the most important predicting variables that determine the frequency of interaction for individual taxonomic groups of flower-visiting insects. To see how changes in interaction, made by flower-visiting insects, tracks flowering plants, the RELATE function in Primer was used to compare the resemblance matrix of flower-visiting insects’ interaction to the resemblance matrix of flowering plants. This function is important for comparing similarity of two sets of multivariate data matrices by calculating the rank correlation coefficient of the element of the two matrices [59]

## Results

A total of 1344 interactions were observed between 71(S1 Table) flower-visiting insect species and 32 (S2 Table) flowering plant species. For all zones combined, interactions consisted of bees (53.5%), beetles (28.5%), wasps (9.1%), and flies (8.9%). This pattern was mostly consistent at each zone separately, with bees making up half of all interactions, except at the peak zone where bee interactions dropped to 36% and beetle interactions increased to 34%.

**Fig 1:**
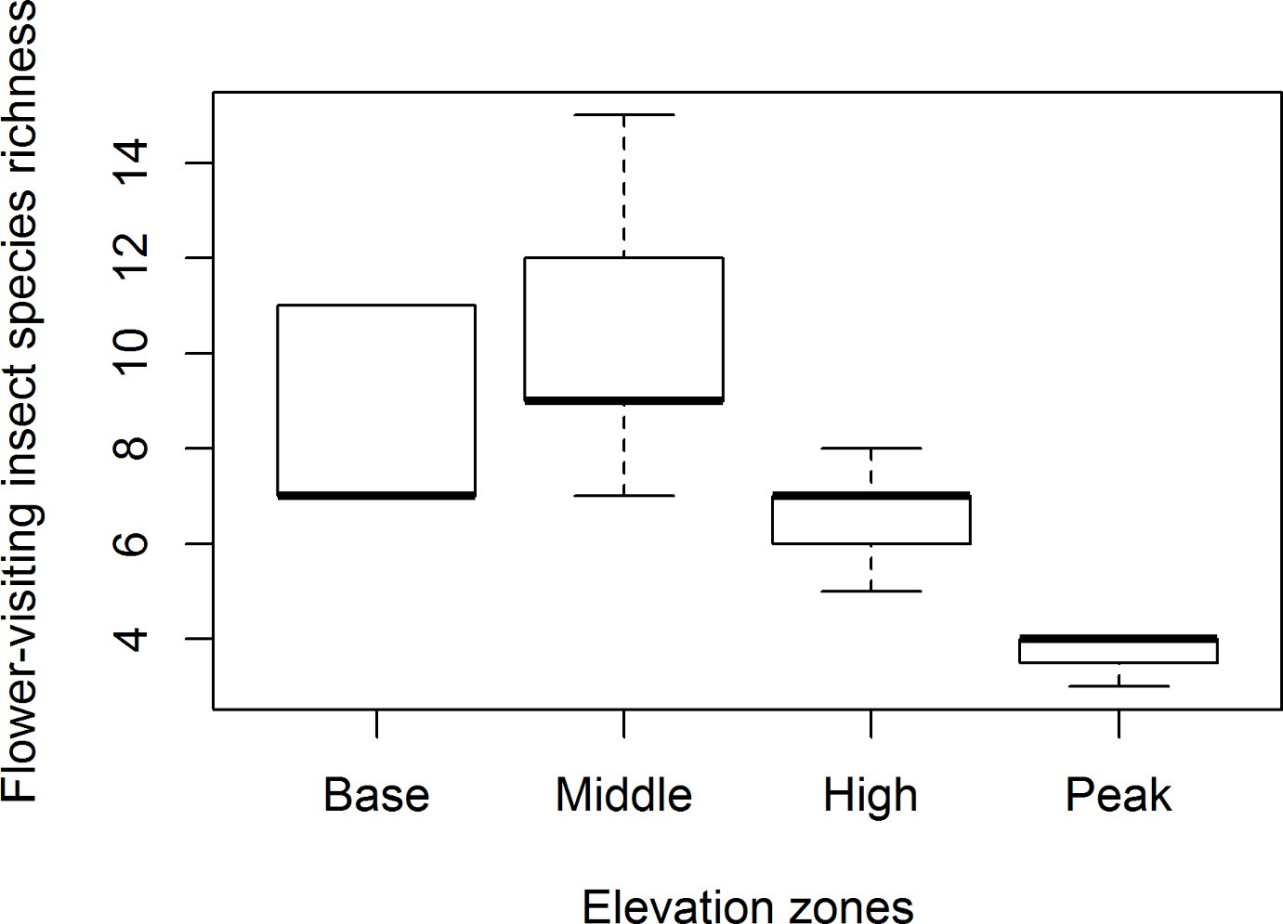
Mean species richness (±SE) of flower-visiting insects across elevation zones. Means with similar letters are not significantly different at P>0.05.

**Fig 2:**
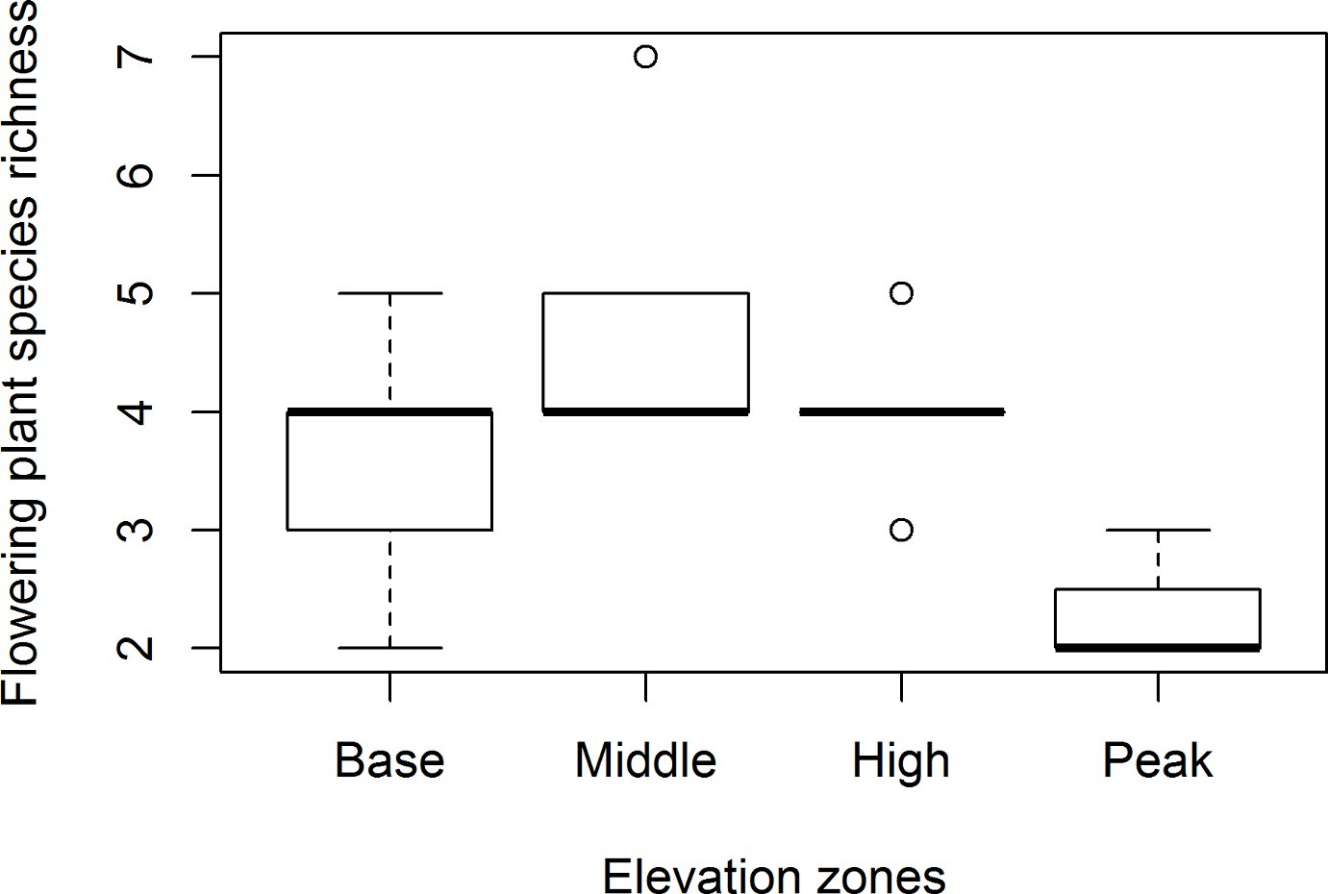
Mean species richness (±SE) of flowering plants across elevation zones. Means with similar letters are not significantly different at P>0.05.

There were significant differences in both flower-visiting insect species and plant species richness across all zones combined. The highest species richness of flower-visiting insects (z = 3.141, P = 0.008, Fig 1) and plants (z = 3.532, P = 0.002, Fig 2) was recorded at the middle zone, and the lowest at the peak zone.

**Fig 3:**
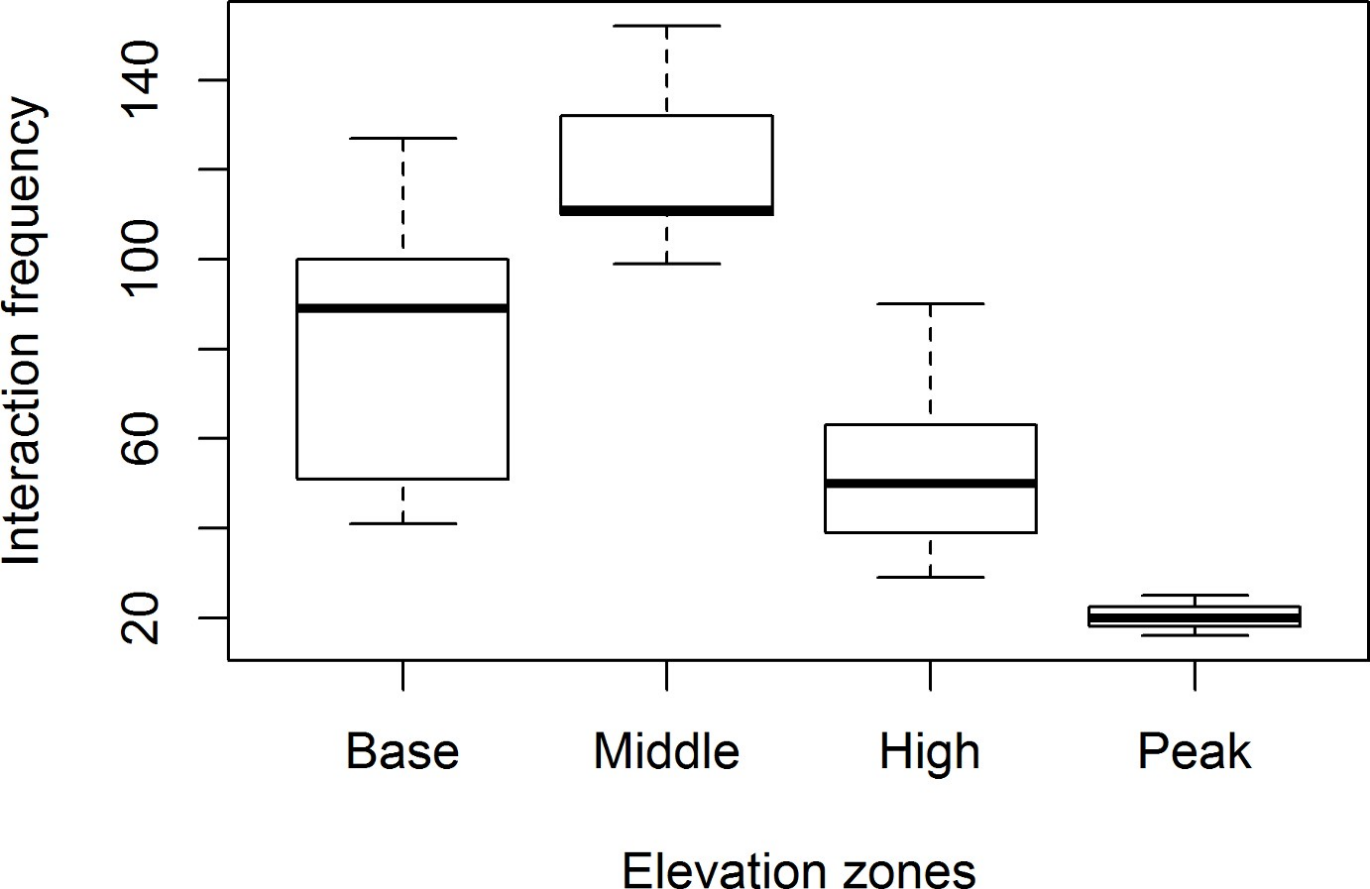
Mean number of interactions (±SE) across elevation zones. Means with similar letters are not significantly different at P>0.05.

**Fig 4:**
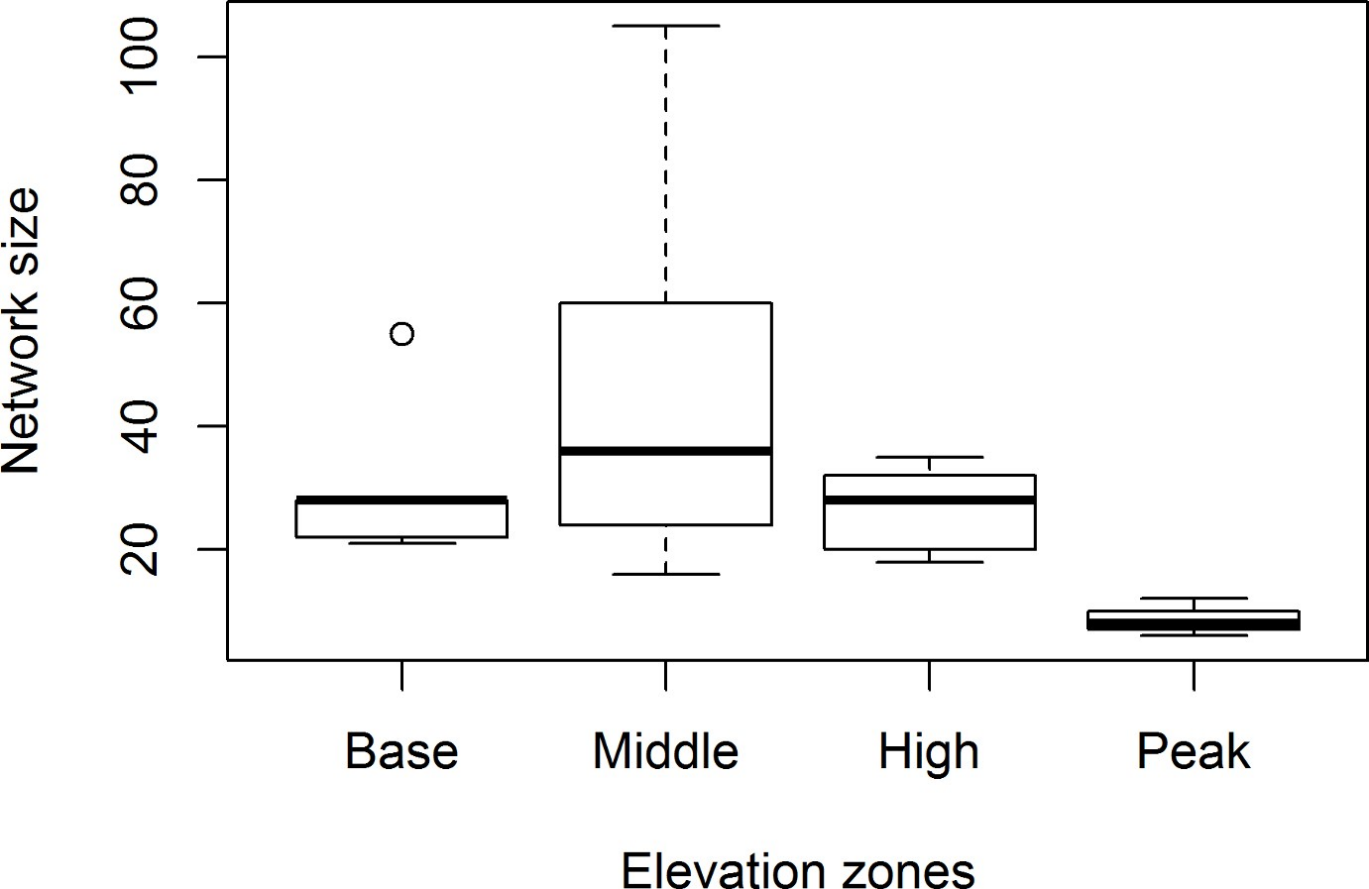
Mean interaction network size (±SE) across elevation zones. Means with similar letters are not significantly different at P>0.05.

There was also a significant difference in frequency of interaction and network size across zones. Highest number of interactions (z = 7.049, P<0.0001, Fig 3) and largest number of networks (z = 4.322, P<0.0001, Fig 4) were in the middle zone, which differed significantly from the small-sized and few interactions in the peak zone.

Most of the network metrics showed no significant differences across zones. However, network linkage density (F_3,14_ = 4.145, P = 0.027, Table 1), network generality (F_3, 14_ = 5.528, P = 0.0101, Table 1), and network Shannon diversity (F_3,14_ = 18.11, P = 0.00004, Table 1) showed significant differences across zones. At the species level, flower visitors were moderately specialized (bee = 0.43±0.03, beetle = 0.45±0.06, fly = 0.32±0.04, wasp = 0.33±0.05). However, mean specialization (d’) index did not differ significantly across zones (F_3,129_ = 0.795, P = 0.499),or among taxonomic groups (F_3,129_ = 1.506, P = 0.216).

**Table 1:** Mean(±SE) of network metrics in the four elevation categories.

	Base zone	Middle zone	High zone	Peak zone	F value	P value
**Connectance**	0.4±0.04	0.37±0.049	0.34±0.041	0.60±0.077	3.308	0.052
**Nestedness**	42.882±5.065	39.801±4.391	30.214±4.562	32.211±4.10	1.681	0.217
**ISA**	0.408±0.089	0.254±0.055	0.220±0.144	0.210±0.112	0.775	0.527
**Shannon diversity**	2.165±0.126	2.525±0.119	1.833±0.074	1.330±0.095	18.11	0.00043
**Generality**	1.419±0.107	1.905±0.149	1.403±0.104	1.244±0.074	5.528	0.01
**Linkage density**	2.516±0.389	2.833±0.200	1.924±0.199	1.529±0.067	4.145	0.027
**Specialisation (H**_**2**_**’)**	0.726±0.057	0.568±0.076	0.799±0.113	0.888±0.028	2.409	0.111

ISA = interaction strength asymmetry.

Overall, insect activity increased significantly with flower diversity. However, this varied among taxonomic groups. Bees (r = 0.449, P = 0.05, Table 2) and beetles (r = 0.482, P = 0.05, Table 2) showed the strongest positive correlation with flower diversity, with wasps showing a negative relationship with flower diversity. The relationship between wasps or beetles and flower diversity was not significant in either case.

**Table 2:** Correlation of visitation frequency to flower diversity and flower area for different insect taxa.

	Flower diversity		Flower area	
	R	P	R	P
**All insects**	0.4773	0.0003	0.4704	0.0004
**Bees**	0.4486	0.05	0.6625	0.003
**Beetles**	0.4816	0.05	0.5022	0.047
**Wasps**	-0.0753	0.847	-0.0251	0.949
**Flies**	0.3781	0.2814	0.3415	0.334

There was a significant positive relationship between flower-visiting insect activity and flower area, although the strength and direction of the relationship varied among insect groups. Bees showed the strongest relationship with flower area (r = 0.663, P = 0.003, Table 2), followed by beetles (r = 0.502, P = 0.047, Table 2). Flies showed no significant relationship, while wasps showed a negative relationship, but this was not significant (Table 2).

### Species separation across zones

The ANOSIM indicated a significant separation in species of flower-visiting insects across zones (R = 0.516, P = 0.001, Table 3). The greatest separation was between insect species at the middle zone and the peak zone (R = 0.968, P = 0.018, Table 3). The degree of separation differed significantly among taxonomic groups. Bees showed the greatest separation (R = 0.454, P = 0.001, Table 3) across zones, followed by beetles (R = 0.25, P = 0.005, Table 3), then wasps (R = 0.115, P = 0.008, Table 3). However, none of the pairwise comparisons was significant. There was no significant separation in fly species across zones (R = 0.034, P = 0.204, Table 3)

**Table 3:** Analysis of Similarity showing pairwise comparison of interaction frequency of insect taxa for elevation categories.

Elevation zones		R	P
**All insects**		**0.516**	**0.001**
Base	Middle	0.478	0.008
Base	High	0.68	0.008
Base	Four	0.41	0.036
Middle	Three	0.236	0.040
Middle	Peak	0.969	0.018
High	Peak	0.559	0.018
**Beetles**		**0.25**	**0.005**
Base	Middle	0.078	0.222
Base	High	-0.002	0.444
Base	Four	0.333	0.054
Middle	Three	0.014	0.373
Middle	Peak	0.846	0.018
High	Peak	0.462	0.036
**Wasps**		**0.115**	**0.008**
Base	Middle	0.105	0.206
Base	High	0.118	0.111
Base	Four	0.1	0.25
Middle	Three	0.125	0.206
Middle	Peak	0.111	0.464
High	Peak	0.143	0.464
**Bees**		**0.454**	**0.001**
Base	Middle	0.568	0.008
Base	High	0.65	0.008
Base	Four	0.462	0.036
Middle	Three	0.22	0.063
Middle	Peak	0.744	0.018
High	Peak	0.251	0.107
**Flies**		**0.034**	**0.204**
Base	Middle	-0.034	0.762
Base	High	0.154	0.167
Base	Four	-0.039	0.607
Middle	Three	0.077	0.286
Middle	Peak	-0.1	0.786
High	Peak	0.143	0.036

**Fig 5:**
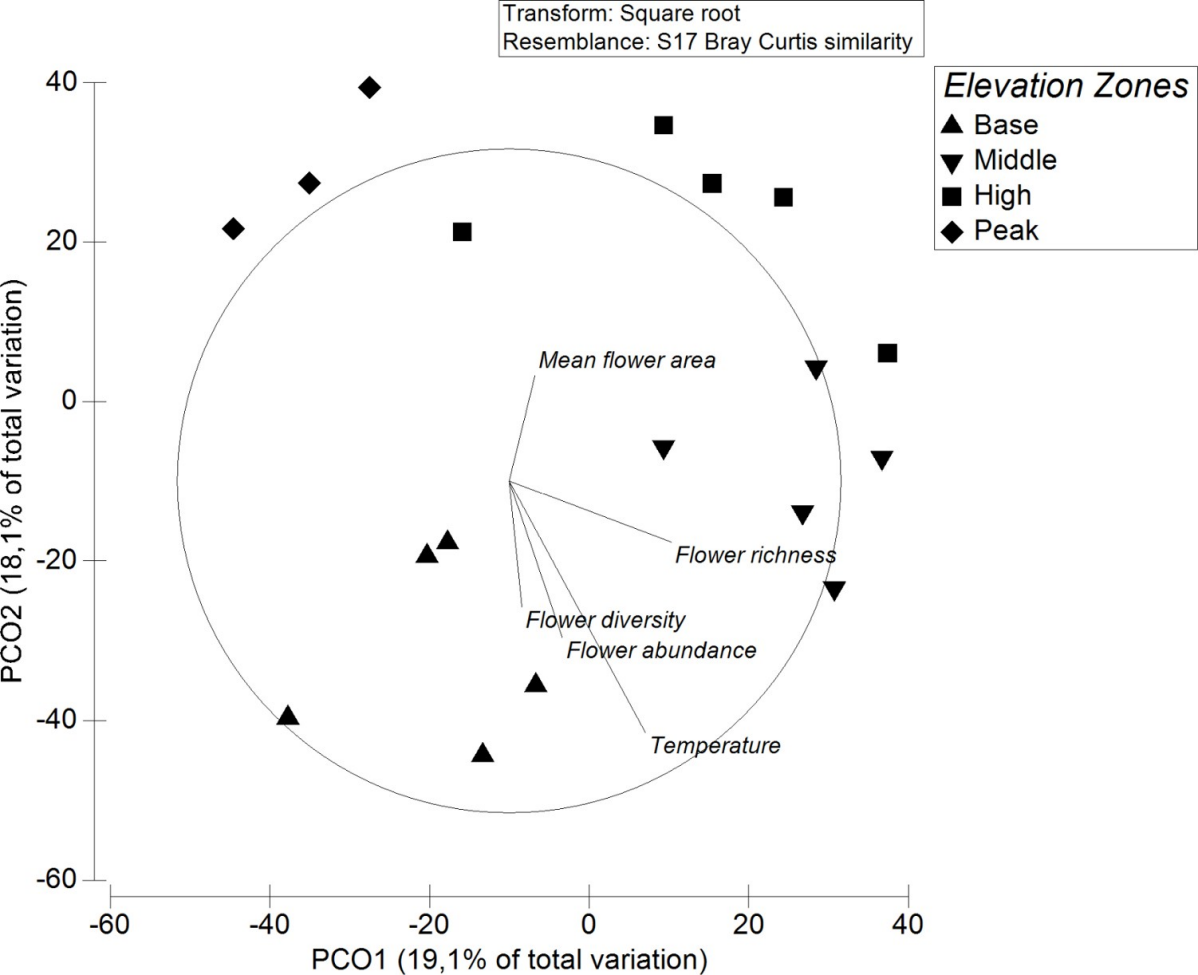
PCO showing separation of insect activities at different elevation zones, and the direction of environmental variables.

The PCO gives a visual representation of the separation among zones, as well as the direction of the environmental variables. Insect activities in the peak zones strongly separated out from those of all other zones (Fig 5). Temperature increased towards the base zone, which was similar to the direction of flow for flower richness, flower diversity, and flower abundance, while flower area increased towards the high zone (Fig 5)

### Effect of environmental variables on flower-visiting insects activities

Of all the explanatory environmental variables in our model, air temperature was the only significant factor driving the pattern of interaction of flower-visiting insects across the elevation gradient with a prediction of 14.4% of total insect activity (Table 4).

**Table 4:** Distlm model showing important predictors of insect visitation frequency among taxonomic group.

	Variable	AIC	SS(trace)	Pseudo-F	P	Prop.	Cumul.	Res.df
**All insects**	Temperature	149.28	9711.3	2.6973	0.001	0.14426	0.14426	16
**Beetles**	Temperature	149.25	1170.6	3.2565	0.002	0.16911	0.16911	16
	Flower area	148.78	7364.9	2.2028	0.031	0.10639	0.27551	15
	Flower abundance	148.06	7051.9	2.2907	0.023	0.10187	0.37738	14
**Bees**	Temperature	148.73	10319	2.9551	0.003	0.1559	0.1559	16

AIC = Alkaike information criterion, SS = Sum of square, Prop. = Percentage variation explained by variable in model, Cumul. = Cummulative percentage variation explained by model, Res.df = Degree of freedom of residuals

Similarly, when we assessed effect of these variables on interaction of different taxonomic groups, air temperature was the only significant factor explaining the variation of bee interactions across the elevation gradient, with a prediction of 15.6% (Table 4). In turn, variation in interactions involving beetles was more strongly influenced by flower abundance and flower area, with estimated predictions of 37.8% and 27.6% respectively, and a moderate prediction of 16.9% in the case of temperature (Table 4). None of the environmental variables in our model showed significant predictive ability for pattern of interaction observed in wasps and flies.

### Relationship between plant composition and insect activities

The RELATE comparison showed a significant relationship between the resemblance matrix of plant composition and activities of flower-visiting insects across all zones (rho = 0.418, p = 0.001).

## Discussion

Bees are important pollinators, with most species being actively dependent on floral resources. The CFR is the only biodiversity hotspot where high plant and bee diversity coincide [43], this explains the dominance of bees in interactions with flowering plants, as seen here. The flower-visiting insect groups here showed a strong relationship with flower area rather than with diversity of flowering plants. In the CFR, plots with high floral density are highly attractive to flower-visiting insects [49]. More compacted inflorescence with wider area of display attracts more flower-visiting insects compared to a single flower head with smaller display area [60]. This explains the stronger relationship observed between insects and flower area here, especially for bees that depend on the floral resources.

There was a significant difference in species richness of flower-visiting insects and flowering plants across the different elevation zones. Insects are usually highly sensitive to fluctuations in environmental factors, including those across different elevations. One of the major factors associated with elevation change is temperature, which influences foraging activities of flower visiting insects [61], and also the productivity of flowering plants [62]. Thus, flower visiting insects composition tracks flowering plant as we found here, and as reported by Winfree et al [63]. This implies that the presence of different species of flowering plants at different zones of elevation in our study supports the distribution of different species of flower-visiting insects across the elevation gradient.

Although quantity of floral resource available in an ecosystem is one of the most important factors driving flower visitation by insects [64], here we show that the species of plant available is also of great importance, especially in a system like this one, with its distinct zonation of flowering plants. This largely supports our second hypothesis. Also, for a mountain like ours, with varying level of environmental stress and a unique distribution of flowering plants, loss of important flower species at any zone may have a direct effect on the displacement of flower-visiting insects across elevations.

### Interaction network properties

Here we show significant changes in some network quantitative indices across elevation zones. There was a significant difference in interaction linkage density across zones. Linkage density, which is the average number of feeding links for a species, is dependent on availability of interaction partners. On our mountain, this decreased at the peak elevation, as also observed on Mt. Wilhelm, Papua New Guinea [37]. The decline in interacting partners at high elevation also drove the pattern of network generality in our study. Highest network generality recorded in the middle zone is indicative of more floral resources, even though flower abundance was not significantly different across zones. Presence of highly rewarding flowering plants like *Lobostemon glaber* and *L*. *trichotomus* in high abundance at the base, middle and high zones may have led to higher generality at these elevations compared to the peak zone. Flowers of this genus are attractive to bees [65, 66] and received high visitation rate in our study. Highest network generality at the middle zone, which is also an ecotone, may be important for the stability of these networks and their resilience to environmental stress. As a link between zones, ecotones are evidence of rapid climatic transitions along gradients, and are important conservation areas in ecosystems [67]. Furthermore, plant species richness was also highest in this zone. More flower resources may encourage selectiveness of flower-visitors, especially for more specialized insect species, and overall will confer stability and robustness to interaction networks [68, 69, 70].

Network diversity accounts for the distribution of interaction frequency among interacting species [71, 72]. Communities rich in interacting partners and former links are usually more stable compared to communities made up of few individual species [73, 74]. Although there has been criticism of the interpretation of network diversity, Dunne, [73] argues that network stability is directly proportional to network diversity, as long as connectance decreases. In our study, although only marginally significant, connectance value decreases with increase in network diversity across zones. This further supports the stability of interaction networks at lower zones in the face of environmental pressures. In contrast, low network diversity at the peak zone illustrated the degree of vulnerability of the networks there to disruption. This also shows how sensitive species are at this elevation, where environmental conditions are extreme in comparison with those at the lower elevations.

### Separation across elevation and effect of temperature

Agenbag et al. [45] showed a distinct separation in flowering plant species communities on our mountain. For the elevation-sensitive species on our mountain such as *P*. *repens*, which prefers high rather than low zones, this plant species may be vulnerable to climate change as warmer and drier conditions begin to prevail in this Mediterranean-type ecosystem [75].

Flower-visiting insect species at the middle zone were very different from those in the peak zone. Bees were the most sensitive to change in elevation, with significantly different species composition at each elevation zone. This suggests huge turnover in interactions among bees, determined mostly by temperature rather than flower indices, as explained by our model selection of environmental variables. In the case of ants, temperature was the major predicting factor for their distribution across a nearby elevation gradient [76]. Body size is important for how bee species tolerate adverse climatic conditions with elevation [39]. Here, we observed *Xylocopa* spp. and moderate-sized Megachilidae spp. across all elevations. However, *Lasioglossum* spp. and other small-sized Halictidae differed in species composition at different elevations. Big-sized Bombyliidae and the beetle *Claniaglenlyonensis* were only recorded at the peak zone, with small-sized monkey beetles only at the lower elevations.

The greatest difference in species interactions was between middle and peak zones. However, there was also a significant difference between the base and middle zones. The middle, ecotonal zone had the highest bee species interactions, as well as highest species richness, indicating the presence of most suitable abiotic conditions necessary for a rich interaction between bees and flowers. It appears that such ecotonal zones in general are rich in species and interactions [77, 78], with already some indications of an upward shift in bumblebee distributions in montane ecosystems [79, 80, 81].

The peak zone here had the fewest interactions involving bees, indicating that it is the least climatically suitable zone for supporting bee-flower interactions. Although temperature was the major predicting factor for local bee elevation distribution, the decline in flower diversity at the peak elevation may also have acted in synergy with temperature to reduce bee diversity [82]

Beetles showed a weaker zonal difference than bees, although there was a highly significant difference in species composition between the lowest two zones and the peak, determined, as with bees, by temperature and flower composition. CFR plant diversity may explain the diversity of insect assemblages better than abiotic factors [83], with monkey beetles being one of the most important pollinator groups for most flowers here [84]. Although bees have highest flower visitation, monkey beetles carry higher pollen loads, at least of Astereacea and Aizocea species [85]. Decline in flower richness and diversity, especially in the peak zone, may be the most important factor, rather than temperature, influencing reducing beetle diversity over the elevation gradient. Nevertheless, plant diversity may have been driven in part by temperature in addition to the underlying factors of soil types, low nutrients, and orographic patterns [45], which in turn, may influence beetle diversity.

We found that flies and wasps were not significantly influenced by temperature or flower indices, and unlike bees, these groups are not obligate nectar feeders, and are less affected by flower abundance and composition [86]. We show here that flies and wasps are less sensitive to changes in environmental factors, and so may be less suitable for monitoring of changes in flower indices and the abiotic effects of elevation.

## Conclusion

As elsewhere, elevation stress here influenced interactions between plants and flower-visiting insects, but with the various insect groups being influenced differentially, by the direct effect of temperature and the indirect effect of flowering plant diversity and area. With an anticipated overall temperature increase in the area of about 1.8^°^C by 2050 [42], our results suggest that the current insect-flower interactions, especially those involving bees, are vulnerable to temperature changes where interactions decline with reduced temperature at peak elevation. This may not necessarily be negative in view of the richer and more robust interactions at the lower elevations where temperature is higher, and the presence of monkey beetles, wasps and flies. However, there is likely to be loss of certain local species that are currently only at the peak elevation such as *Clania glenlyonensis* and *Bombyliidae* sp2. Also, with flowering plants driving insect composition across the elevation gradient, loss of flowering plant species unique to any of the zones, especially the peak zone where very few flowering plant species are available, may eventually result in a local displacement of visiting insects on this mountain. Finally, we provide here some data against which interaction networks could be compared in the future.

## Supporting information

S1 Table List of flower-visiting insect species. (DOCX)

S2 Table List of plant species. (DOCX)

S1 Data Data.zip. Network data for insect-flower interactions across elevation zones.
(ZIP)
