# Supplementary material for: Insect-flower interaction networks vary among endemic pollinator taxa over an elevation gradient
Source: PLoS One. 2018 Nov 29;13(11):e0207453. doi: 10.1371/journal.pone.0207453 (PMC6264482; doi:10.1371/journal.pone.0207453)
Supplement: S1 Table — (DOCX) [file pone.0207453.s001.docx]

**S1 Table. List of flower-visiting insect species**

| Taxa | Family | Genus | Species/Morphospecies |
| --- | --- | --- | --- |
| Bee | Halictidae | *Allodapula* | *melanopus* |
| Bee | Halictidae | *Halictus* | sp1 |
| Bee | Halictidae | *Halictus* | sp2 |
| Bee | Halictidae | *Halictus* | sp3 |
| Bee | Halictidae | *Halictus* | sp4 |
| Bee | Halictidae | *Halictus* | sp5 |
| Bee | Halictidae | *Halictus* | sp6 |
| Bee | Halictidae | *Halictus* | sp7 |
| Bee | Halictidae | *Lassioglosum* | sp1 |
| Bee | Halictidae | *Lassioglosum* | sp2 |
| Bee | Colletidae | *Colletes* | sp1 |
| Bee | Colletidae | *Colletes* | sp2 |
| Bee | Colletidae | *Colletes* | sp3 |
| Bee | Colletidae | *Colletes* | sp4 |
| Bee | Colletidae | Colletidae | sp |
| Bee | Megachilidae | *Megachile* | sp1 |
| Bee | Megachilidae | *Megachile* | sp2 |
| Bee | Megachilidae | *Megachile* | sp3 |
| Bee | Anthophoridae | *Amegilla* | sp |
| Bee | Anthophoridae | *Anthophora* | sp1 |
| Bee | Anthophoridae | *Anthophora* | sp2 |
| Bee | Anthophoridae | *Anthophora* | sp3 |
| Bee | Anthophoridae | *Anthophora* | sp4 |
| Bee | Apidae | *Apis* | *melifera* |
| Bee | Apidae | Apidae | sp1 |
| Bee | Apidae | Apidae | sp2 |
| Bee | Apidae | *Xylocopa* | *olivacea* |
| Bee | Apidae | *Xylocopa* | sp1 |
| Bee | Apidae | *Xylocopa* | sp2 |
| Bee | Apidae | *Xylocopa* | sp3 |
| Bee | Apidae | *Xylocopa* | sp5 |
| Bee | Apidae | *Xylocopa* | sp6 |
| Bee | Apidae | Apidae | sp3 |
| Bee | Apidae | *Tetralonia* | sp1 |
| Bee | Apidae | *Tetralonia* | sp2 |
| Bee | Ceratinidae | Ceratinidae | sp |
| Beetle | Cerambycidae | Cerambycidae | sp1 |
| Beetle | Cerambycidae | Cerambycidae | sp2 |
| Beetle | Cerambycidae | Cerambycidae | sp3 |
| Beetle | Cerambycidae | Cerambycidae | sp4 |
| Beetle | Cerambycidae | *Typocerus* | sp |
| Beetle | Scarabaeidae | *Heterochelus* | sp |
| Beetle | Scarabaeidae | *Hedybius* | sp |
| Beetle | Scarabaeidae | *Peritrichia* | sp1 |
| Beetle | Scarabaeidae | *Peritrichia* | sp2 |
| Beetle | Scarabaeidae | *Peritrichia* | sp3 |
| Beetle | Scarabaeidae | *Clania* | *glenlyonensis* |
| Beetle | Scarabaeidae | *Anisomyx* | *ursus* |
| Beetle | Scarabaeidae | *Anisomyx* | sp |
| Fly | Muscidae | Muscidae | sp1 |
| Fly | Muscidae | Muscidae | sp2 |
| Fly | Syrphidae | Syrphidae | sp1 |
| Fly | Syrphidae | Syrphidae | sp2 |
| Fly | Syrphidae | Syrphidae | sp3 |
| Fly | Bombylidae | Bombylidae | sp1 |
| Fly | Bombylidae | Bombylidae | sp2 |
| Fly | Bombylidae | Bombylidae | sp3 |
| Fly | Bombylidae | Bombylidae | sp4 |
| Fly | Caliphoridae | Caliphoridae | sp |
| Fly | Culicidae | Culicidae | sp |
| Wasp | Sphecidae | Sphecidae | sp1 |
| Wasp | Sphecidae | Sphecidae | sp2 |
| Wasp | Sphecidae | Sphecidae | sp3 |
| Wasp | Sphecidae | Sphecidae | sp4 |
| Wasp | Vespidae | Vespidae | sp1 |
| Wasp | Vespidae | Vespidae | sp2 |
| Wasp | Eumenidae | Eumenidae | sp1 |
| Wasp | Eumenidae | Eumenidae | sp2 |
| Wasp | Pompilidae | Pompilidae | sp1 |
| Wasp | Pompilidae | Pompilidae | sp2 |
| Wasp | Icheumonidae | Ichneumonidae | sp |
