# Supplementary material for: Insect-flower interaction networks vary among endemic pollinator taxa over an elevation gradient
Source: PLoS One. 2018 Nov 29;13(11):e0207453. doi: 10.1371/journal.pone.0207453 (PMC6264482; doi:10.1371/journal.pone.0207453)
Supplement: S2 Table — (DOCX) [file pone.0207453.s002.docx]

**S2 Table. List of plant species**

| Family | Genus | Species |
| --- | --- | --- |
| Aizoceae | *Ruschia* | *tecta* |
| Asteraceae | *Arctotis* | *gousblom* |
| Asteraceae | *Oedera* | *squarrosa* |
| Asteraceae | *Helichrysum* | *dasyanthum* |
| Asteraceae | *Oncosiphon* | *grandiflorum* |
| Asteraceae | *Cullumia* | sp1 |
| Asteraceae | *Cullumia* | sp2 |
| Asteraceae | *Senecio* | sp1 |
| Asteraceae | *Senecio* | sp2 |
| Asteraceae | *Senecio* | sp3 |
| Asteraceae | *Heterolepis* | *aliena* |
| Asteraceae | *Dimorphotheca* | *nudicaulis* |
| Asteraceae | *Othonna* | *bulbosa* |
| Asteraceae | *Metalasia* | *muricata* |
| Boraginaceae | *Lobostemum* | *glaber* |
| Boraginaceae | *Lobostemum* | *dorotheae* |
| Boraginaceae | *Lobostemum* | *trichotomus* |
| Geraniacea | *Pelagonium* | sp |
| Malvaceae | *Hermannia* | *alnifolia* |
| Montiniaceae | *Montinia* | sp |
| Montiniaceae | *Montina* | sp2 |
| Polygalaceae | *Polygala* | *fruticosa* |
| Polygalaceae | *Muraltia* | sp |
| Proteaceae | *Protea* | *repens* |
| Proteaceae | *Protea* | *laurifolia* |
| Proteaceae | *Leaucadendron* | *laureolum* |
| Proteaceae | *Leucadendron* | *salignum* |
| Proteaceae | *Erica* | sp1 |
| Proteaceae | *Erica* | sp2 |
| Proteaceae | *Erica* | sp3 |
| Rhamnaceae | *Phylica* | *ericoides* |
| Fabaceae | *Acacia* | sp |
